# Secondary cell wall composition and candidate gene expression in developing willow (*Salix purpurea*) stems

**DOI:** 10.1007/s00425-014-2034-1

**Published:** 2014-02-07

**Authors:** Yongfang Wan, Cristina Gritsch, Theodora Tryfona, Mike J. Ray, Ambrose Andongabo, Keywan Hassani-Pak, Huw D. Jones, Paul Dupree, Angela Karp, Peter R. Shewry, Rowan A. C. Mitchell

**Affiliations:** 1 Rothamsted Research, Harpenden, Hertfordshire AL5 2JQ UK; 2 Biochemistry Department, University of Cambridge, Tennis Court Road, Cambridge, CB2 1QW UK; 3 Department of Life Sciences, Imperial College London, South Kensington Campus, London, SW7 2AZ UK

**Keywords:** Hemicellulose, LM10 antibody, Secondary xylem, Tree stem transcriptome

## Abstract

The properties of the secondary cell wall (SCW) in willow largely determine the suitability of willow biomass feedstock for potential bioenergy and biofuel applications. SCW development has been little studied in willow and it is not known how willow compares with model species, particularly the closely related genus Populus. To address this and relate SCW synthesis to candidate genes in willow, a tractable bud culture-derived system was developed in *Salix purpurea*, and cell wall composition and RNA-Seq transcriptome were followed in stems during early development. A large increase in SCW deposition in the period 0–2 weeks after transfer to soil was characterised by a big increase in xylan content, but no change in the frequency of substitution of xylan with glucuronic acid, and increased abundance of putative transcripts for synthesis of SCW cellulose, xylan and lignin. Histochemical staining and immunolabeling revealed that increased deposition of lignin and xylan was associated with xylem, xylem fibre cells and phloem fibre cells. Transcripts orthologous to those encoding xylan synthase components IRX9 and IRX10 and xylan glucuronyl transferase GUX1 in Arabidopsis were co-expressed, and showed the same spatial pattern of expression revealed by in situ hybridisation at four developmental stages, with abundant expression in proto-xylem, xylem fibre and ray parenchyma cells and some expression in phloem fibre cells. The results show a close similarity with SCW development in Populus species, but also give novel information on the relationship between spatial and temporal variation in xylan-related transcripts and xylan composition.

**Electronic supplementary material:**

The online version of this article (doi:10.1007/s00425-014-2034-1) contains supplementary material, which is available to authorized users.

## Introduction

Plant biomass has potential as a major renewable source of energy (bioenergy) and transport fuels (biofuels). Non-food crops (such as woody crops and perennial grasses) could contribute sustainable feedstocks, yielding greenhouse gas reductions and minimal conflict with food production (Smith and Searchinger 2012). Willows (*Salix* spp.), grown as short rotation coppice, are among the leading commercially grown biomass trees in temperate regions (Karp et al. 2011). They are highly diverse and the *circa* 400 species are broadly grouped into three main subgenera: the tree willows (sub-genus *Salix*), the dwarf and alpine willows (sub-genus *Chamaetia*) and the shrubby willows (sub-genus *Vetrix*). Of these, the shrubby willows are the most suited for biomass due to their propensity for fast, vigorous growth in coppicing cycles, low fertilisation requirements (an average of 20–30 kg N ha^−1^ year^−1^) and ease of vegetative propagation. Breeding of willows for biomass only began in the late 1980s and has focussed on increasing yield for the heat and power industries. More recently, willows have been considered as a potential feedstock source for biofuels. Improvement of willow as a biofuel crop requires an improved understanding of the composition of willow wood and secondary cell walls (SCW), since the sugars needed for conversion for biofuels are derived from lignocellulose.

Willow biomass typically contains around 25 % lignin, 30 % cellulose and 45 % hemicellulose but composition varies. An analysis of 35 field grown willow genotypes, many of which are commercially grown, showed significant variation in the contents of glucose (i.e. cellulose), xylose, arabinose and lignin but not in the contents of mannose and galactose. Interestingly, no clear relationship was found between lignin content and the accessibility of the glucan to enzymatic saccharification (Ray et al. 2012). A study of 138 genotypes from a large mapping population also showed variation in enzymatic glucose release, allowing four enzyme-derived glucose QTLs to be mapped (Brereton et al. 2010). Saccharification yield was found to be independent of biomass yield in this study. However, a beneficial saccharification effect was associated with willow tension wood (Brereton et al. 2011) and recent findings suggest that genotypic differences in response to making tension wood may be a primary determinant of the variation observed in sugar release from willow biomass (Brereton et al. 2012). Taken together, these findings suggest that it will be possible to select for SCW characteristics beneficial to the biofuel process in breeding.

Molecular understanding of the genes controlling SCW properties is critical in identifying causative alleles underlying QTLs and in providing candidate genes for reverse genetic approaches to improve biofuel properties of willow. Of particular interest are genes for synthesis of the polysaccharide xylan which dominates SCW hemicellulose. Xylan is the second most abundant polysaccharide in wood and, as part of the hemicellulosic matrix forming an interface between cellulose microfibrils and hydrophobic lignin, is likely a key determinant of feedstock properties, such as ease of saccharification. It has been shown that disruption of xylan synthesis in poplar (a species closely related to willow) allowed greater cellulose release by cellulase treatment (Lee et al. 2011). The structure of angiosperm wood (hardwood) xylan is similar to that in Arabidopsis, with a backbone of β-(1,4)-linked d-xylose (Xyl) decorated with *O*-2-linked glucuronic acid (GlcA) or methyl-glucuronic acid (MeGlcA) and can be extensively acetylated (Ebringerova et al. 2005) (Supplementary Fig. S1). It also has the characteristic tetrasaccharide at the reducing end β-d-Xyl*p*-(1 → 3)-α-l-Rha*p*(1 → 2)-α-d-Gal*p*A-(1 → 4)-d-Xyl*p* (Pena et al. 2007). Studies of Arabidopsis mutants have led to the identification of candidate genes responsible for all these features, with genes in the glycosyl transferase (GT) families GT8 (IRX8, PARVUS) and GT47 (IRX7) being required for the reducing end tetrasaccharide synthesis, genes in GT43 (IRX9, IRX14) and GT47 (IRX10) families being implicated in backbone synthesis (Brown et al. 2007, 2009; Pena et al. 2007; Wu et al. 2009) and GT8 genes (GUX1, GUX2) being responsible for the addition of GlcA decoration (Mortimer et al. 2010; Rennie et al. 2012). More recently, genes in the DUF579 family have been found to be involved in xylan chain length (Brown et al. 2011; Jensen et al. 2011) and related proteins (GXM) have been shown to be responsible for GlcA methylation (Lee et al. 2012; Urbanowicz et al. 2012). A member of the trichome birefringence-like family (TBL29) has been implicated in xylan *O*-acetylation (Xiong et al. 2013). Homologues of some of these genes have been shown to have equivalent roles in poplar; PoGT47C complements xylan deficits in the *irx7* mutant (Zhou et al. 2006), PoGT8D similarly complements *irx8* (Zhou et al. 2007), and PtrGT43A/B/E and PtrGT43C/D are functional orthologues of IRX9 and IRX14, respectively (Lee et al. 2011).

To relate SCW synthesis to candidate genes in willow, we developed a tractable system, based on re-growth from axillary buds, to study composition and gene expression of developing willow stems in a standardised, controlled way. Using this system, we have related both temporal and spatial variation in SCW composition to gene expression, focusing on the synthesis of xylan and compared our findings with those from other model systems.

## Materials and methods

### Bud isolation and plantlet culture

Willow stems (*Salix purpurea* L. genotype 844: Rothamsted National Willow Collection) were harvested from established plants. Actively growing stems of approximately 5–10 mm diameter were cut into 25 cm lengths and surface-sterilised in 70 % (v/v) aq. ethanol for 3 min followed by 10 % (v/v) sodium hypochlorite for 3 min. After three washes with sterile water, the axillary buds were cut from the stem, the bud scales and stem epidermis removed under a dissecting microscope, and the isolated buds placed upright on plates containing root induction medium [1/2 strength MS salts, 1× MS vitamins, 0.2 mg l^−1^ indole-3-butyric acid (IBA), 100 mg l^−1^ myo-inositol, 20 g l^−1^ sucrose, solidified with 6 g l^−1^ phytagel]. After 4–6 weeks growth at 22–24 °C, the roots were 1–2 cm long and the plants were transferred to root induction medium (but without IBA) for another 10 days and then transplanted to soil and moved to a controlled environment cabinet at 20/15 °C, 14/10 h (day/night) and 330 μmol m^−2^ s^−1^ light. The third stem internodes (counting from the basal stem) were collected at week 0 (before being transplanted to soil) and weeks 2, 4, and 6 (after transplantation to soil) and were either analysed immediately or frozen into liquid nitrogen. To obtain sufficient material for analyses, multiple internodes were pooled into samples (~70, ~50, ~20, 8 internodes at 0, 2, 4 and 6 weeks, respectively).

### Alcohol insoluble residue (AIR) preparation and enzyme hydrolysis

Willow stems were harvested, milled, submerged in 96 % (v/v) ethanol, and boiled at 70 °C for 30 min to inactivate enzymes. Following homogenisation using a ball mill (Glen Creston), the pellet was collected by centrifugation (4,000*g* for 15 min). The pellet was then washed with 100 % (v/v) ethanol, twice with chloroform:methanol (2:1 v/v), followed by successive washes with 65, 80 and 100 % (v/v) ethanol. The remaining AIR pellet was air dried at 70 °C overnight. Aqueous suspensions of AIR were prepared using a glass homogeniser. 100 μg of AIR was treated with 20 μl 4 M NaOH for 1 h and neutralised with HCl. Xylan was digested in 0.1 M ammonium acetate buffer (pH 5.5) overnight at 21 °C with excess of enzyme before boiling for 30 min to inactive enzymes. *Cellvibrio mixtus* xylanase Xyn10B from glycosyl hydrolase family 10 was a kind gift from Prof. Harry Gilbert (Newcastle University, Newcastle upon Tyne, UK). After digestion, samples were dried in vacuo and labelled with 8-aminopyrene-1,3,6-trisulfonic acid (APTS) for DASH analysis.

### Quantification of oligosaccharides by DNA-sequencer assisted saccharide high throughput analysis (DASH)

The Xyn10B hydrolysis products were reductively aminated with APTS (Biotium), together with appropriate controls to determine non-specific bands, as described by Li et al. (2013). Following derivatisation, the oligosaccharides were diluted to 1 μg ml^−1^ initial AIR. 20 μl of hydrolysed material was loaded onto a 96-well plate together with spectrally distinct mobility standards, prepared as described by Li et al. (2013). Samples were dried in vacuo and resuspended in 20 μl Hi-Di formamide (Applied Biosystems). Hydrolysis products were separated by capillary electrophoresis on a 3730xl DNA Analyser (Applied Biosystems) and data analysed using in house ‘DASHboard’ software (Li et al. 2013). Traces were aligned according to their fractional mobility with respect to the electrophoretic mobility markers. For the calculation of branching frequency, data from Xyn10B were used to estimate the ratio of [Me]GlcA to Xyl in the xylanase-released oligosaccharides.

### RNA extraction

The RNA extraction method was based on Chang et al. (1993). Frozen tissues were ground in liquid nitrogen and extracted in hexadecyltrimethylammonium bromide (CTAB) buffer [2 % CTAB, 2 % polyvinylpyrrolidone K30, 100 mM Tris/HCl, pH 8.0, 25 mM ethylene diaminetetra-acetic acid (EDTA), 2.0 M NaCl, 0.5 g l^−1^ spermidine, 2 % (w/v) 2-mercaptoethanol] with chloroform/isoamyl alcohol (24:1 v/v) to remove proteins. RNA was precipitated with 10 M LiCl and incubated on ice overnight, dissolved in buffer [1.0 M NaCl, 0.5 % (w/v) sodium dodecyl sulphate, 10 mM Tris/HCl pH 8.0, 1 mM EDTA] to remove polysaccharides and extracted once with chloroform/IAA (24:1 v/v). After ethanol precipitation, the total RNA was dissolved in diethylpyrocarbonate (DEPC)-treated water and stored at −80 °C.

### Sample preparation for microscopy

All solutions were prepared using DEPC-treated H_2_O and aseptic procedures were followed to prevent contamination and loss of RNA from samples. Transverse discs (1–3 mm thick) were cut from the middle of internode 3 in fixative [4 % (w/v) paraformaldehyde in 0.1 M Sorensen’s phosphate buffer (prepared with NaH_2_PO_4_·2H_2_O and Na_2_HPO_4_·12H_2_O buffer, pH 7)]. At week 0, the internode below the first set of completely opened leaves below the tip was used. Samples were fixed for 4 h at RT and then overnight at 4 °C. After several washes in buffer, the tissues were dehydrated in an ethanol series and embedded in either LR White medium grade resin (London Resin, TAAB) or paraffin wax (Paraplast Plus, Sigma). Resin sections were cut with a Reichert–Jung ultramicrotome at 1 μm thickness, stained with Toluidine Blue O [0.01 % (w/v) toluidine blue in 1 % (v/v) sodium tetraborate, pH 9] and permanently mounted on glass slides with DPX mountant (Sigma) for general morphological observations. Wax-embedded material was sectioned at 10–12 μm thick using a Reichert rotary microtome. Sections were floated on DEPC-treated H_2_O at 40 °C, collected onto polysine slides, dried on a hot plate at 40 °C and incubated in an oven at 37 °C overnight to ensure complete drying. Slides were kept at 4 °C until required. These were used for in situ hybridization experiments and lignin staining. The phloroglucinol–HCl staining method was used to identify lignified tissues. Sections were de-waxed in Histo-Clear (Sigma) and rehydrated in an ethanol series. After air drying, the sections were treated with phloroglucinol solution [3 % (w/v) in 95 % (v/v) ethanol] for 1 min, followed by a drop of HCl and mounted in glycerol for immediate examination. Observations were made with a Zeiss Axiophot upright microscope using bright-field optics. A Retiga Exi CCD digital camera (Qimaging, Surrey, BC, Canada) and MetaMorph software (version 7.5.5, Molecular Devices, Sunnyvale, CA, USA) were used to acquire the images.

### Immunolabeling and confocal microscopy

Resin sections (1 μm thick) collected on multiwell slides coated with poly-l-lysine hydrobromide (Sigma) were briefly rinsed with PBS and incubated in blocking solution [PBS, 3 % (w/v) BSA (bovine serum albumin, Sigma A7638), 0.1 % (w/v) Tween 20] for 1 h. This was followed by 2-h incubation in rat monoclonal LM10 (PlantProbes) antibody (for unsubstituted or low-substituted xylans) diluted 1:50 in 1 % (w/v) BSA in PBST (PBS, 0.1 % Tween 20) at RT. After several washes in PBST, the sections were incubated for 1 h at RT in the dark with anti-rat Alexa Fluor 633 (Invitrogen) secondary antibody. The 633 fluorochrome was chosen, as at this wavelength there is little or no autofluorescence from cell walls. After several washes with PBST, PBS and distiled H_2_O, the sections were examined with a Zeiss 780LSM confocal microscope. The images were captured in Z-stack series ranging from 6 to 16 μm and are displayed as maximum intensity projections.

### Probe labelling

The DNA probes were synthesised using PCR primers (Table 1) and were purified and used for in vitro transcription to synthesise and label the RNA probes using DIG labelling kit (Roche, Basel, Switzerland) following the manufacturer’s protocol. All probes were sequenced by Eurofins MWG Operon (London, UK) prior to RNA probe synthesis. The transcribed RNA was hydrolysed in carbonate buffer for 20–30 min, precipitated in ethanol, and dissolved in DEPC water. 1:100 diluted probes (2.6–3.6 μg ml^−1^) were used for hybridisation. The probe efficiency was also tested by spotting the RNA probe onto positively charged nylon membrane with anti-DIG alkaline phosphatase-conjugated antibody and NBT/BCIP colour development.

**Table 1:** Primers for DNA in situ probes synthesis

Oligo name	Sequence^a^	Product size (bp)	
IRX10-AS-F	TCCTCCAATTGGCCTTACTG	730	Antisense probe
IRX10-AS-T7	GAATTGTAATACGACTCACTATAGGGGTGGAAAGCATCACCTGGTT^a^		
IRx10-SP-T7	GAATTGTAATACGACTCACTATAGGGGAAGGACCCCAGATGTCTCA	730	Sense probe
IRX10-SP-R	GTGGAAAGCATCACCTGGTT		
IRX9-AS-F	GGTGCTTAGAAAGACAGGCATTAT	474	Antisense probe
IRX9-AS-T7	AATTGTAATACGACTCACTATAGGGTCATCCTCAAGAGCTACTTGTTTG		
IRX9-SP-T7	GAATTGTAATACGACTCACTATAGGGGGTGCTTAGAAAGACAGGCATTAT	474	Sense probe
IRX9-SP-R	TCATCCTCAAGAGCTACTTGTTTG		
Gux-AS2-F	TGCTCATGAAAAGTGGTGGA	301	Antisense probe
Gux-AS-T7	GAATTGTAATACGACTCACTATAGGGCACAGGCCAGAAAGAGATGC		
Gux-SP-F7	GAATTGTAATACGACTCACTATAGGGTGCTCATGAAAAGTGGTGGA	301	Sense probe
Gux-SP-R	CACAGGCCAGAAAGAGATGC		

^a^Underlined sequence for T7 transcription site

### In situ hybridization

Hybridization was based on Drea et al. (2005) with slight modification. The tissue was de-waxed in Histo-Clear, rehydrated in an ethanol series, digested with Proteinase K for 30 min at 37 °C and post-fixed in 4 % (w/v) paraformaldehyde in PBS (phosphate-buffered saline, pH 7.4) for 10 min. The sections were acetylated for 10 min in 0.1 M triethanolamine buffer with 0.5 % (v/v) acetic anhydride, dehydrated in an ethanol series, and hybridised with the digoxigen (DIG)-labelled RNA probes at 45–50 °C overnight. After post-hybridization washes in 1× SSC (saline sodium citrate buffer) and NTE buffer (2 mM EDTA, 0.5 M NaCl, 1 mM Tris/HCl) at 50 °C, the sections were treated with RNase A at 37 °C for 30 min, and after further stringent washes, incubated in 1 % (w/v) blocking reagent (Roche) for 1 h. Finally, the sections were incubated with anti-DIG alkaline phosphatase antibody conjugate (Roche) diluted 1:1,600. The signal was detected with NBT/BCIP (nitro blue tetrazolium chloride/5-bromo-4-chloro-3-indolyl phosphate toluidine salt, Roche). A Zeiss Axiophot light microscope equipped with a Retiga Exi CCD digital camera and MetaMorph software version 7.5.5 (Molecular Devices) was used to acquire the images under bright-field optics.

### Transcriptomics

RNA-seq was carried out on four samples from third internodes isolated at 0, 2, 4 and 6 weeks after transfer to soil. Library preparation and sequencing were carried out by the University of Bristol Transcriptomics Facility (Bristol, UK) using an Illumina GAiix sequencer generating 114 bp single-end reads. The libraries each generated 10–15 million reads and were quality trimmed and filtered (fastq_quality_trimmer-t 20-l 40), which discarded on average 23 % of the reads. The processed reads were mapped to the complete set of poplar genes (CDS v2.2) using TimeLogic^®^ Tera-BLASTN™ algorithm (Active Motif Inc., Carlsbad, CA, USA) and taking the top-hit with *e* value <1E−20. There were 6.1, 7.0, 5.2 and 5.5 million of these mapped reads for the libraries from 0, 2, 4 and 6 weeks, respectively. Transcript abundance corresponding to any given poplar gene is given by number of reads mapped to this gene per million mapped reads for this library (ppm). Gene families were assigned to poplar genes based on the CAZy database (Cantarel et al. 2009) (for glycosyl transferases) or PFAM domains as listed on Phytozome 9.0 for other gene families (Goodstein et al. 2012).

To look for willow-specific transcripts, the RNA-seq libraries were pooled and de novo assembled using Trinity (Grabherr et al. 2011) (version trinityrnaseq_r2013_08_14). In total 81,701 contigs were obtained and aligned (Tera-BLASTN™, *E* value <1e−5) to poplar CDS sequences. The 22,286 contigs (27.3 %) with no match in poplar were deemed willow-specific and contained ~35,000 ORFs (identified by EMBOSS getorf) potentially encoding peptides of >30 amino acids; HMMER 3.1b1 was then used to search for PFAM domains in these.

## Results

### Development of a tractable study system in willow

**Fig. 1:**
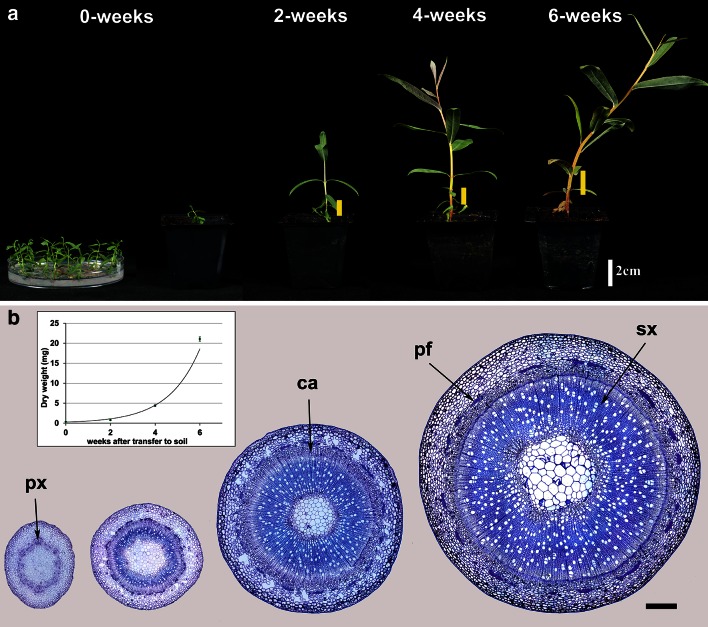
Developmental stages of *Salix purpurea*. **a** Typical plants at the four developmental stages (0, 2, 4 and 6 weeks). Petri dish shows plantlets at the time of transfer to soil. *Yellow lines* and *arrow* in the *inset* image (0-week stage) indicate the position of internode 3. **b** Resin sections stained with Toluidine blue O showing the anatomical development of internode 3 at the same stages. *px* protoxylem, *ca* cambium, *pf* phloem fibres, *sx* secondary xylem. *Bar* 250 μm. *Inset*: dry weight of internode 3. *Line* is fitted exponential curve, *y = * 0.26.e^0.71x^, *R* ^2^ = 0.98

When incubated on solid medium, axillary buds from stems of *S. purpurea* sprouted to give rise to plantlets which, when transferred to soil, grew reproducibly in terms of increases in fresh and dry weight, height and number of internodes. This “bud culture” system was established routinely to allow the initiation and progress of wood formation to be followed in a selected internode in a standardised way. After transplanting to soil, the plantlets elongated very rapidly from about 1 cm to around 25 cm in height, with the mean dry weight of internode 3 increasing from 0.7 to 63 mg (Fig. 1a). The anatomical development of the stem at internode 3 is shown in sections stained with Toluidine Blue in Fig. 1b. The formation of vascular tissues was well underway even at week 0 (date of first transfer to soil) with the first few vessels already having been formed. Between weeks 2 and 6, the cambial zone was composed of several layers of actively dividing cells leading to a rapid increase in the diameter of the stem due to the area of secondary xylem, phloem and phloem fibres. Internode 3 (counting from the base of the stem) was selected to study changes in cell wall synthesis and composition over this developmental period.

### Cell wall composition

**Fig. 2:**
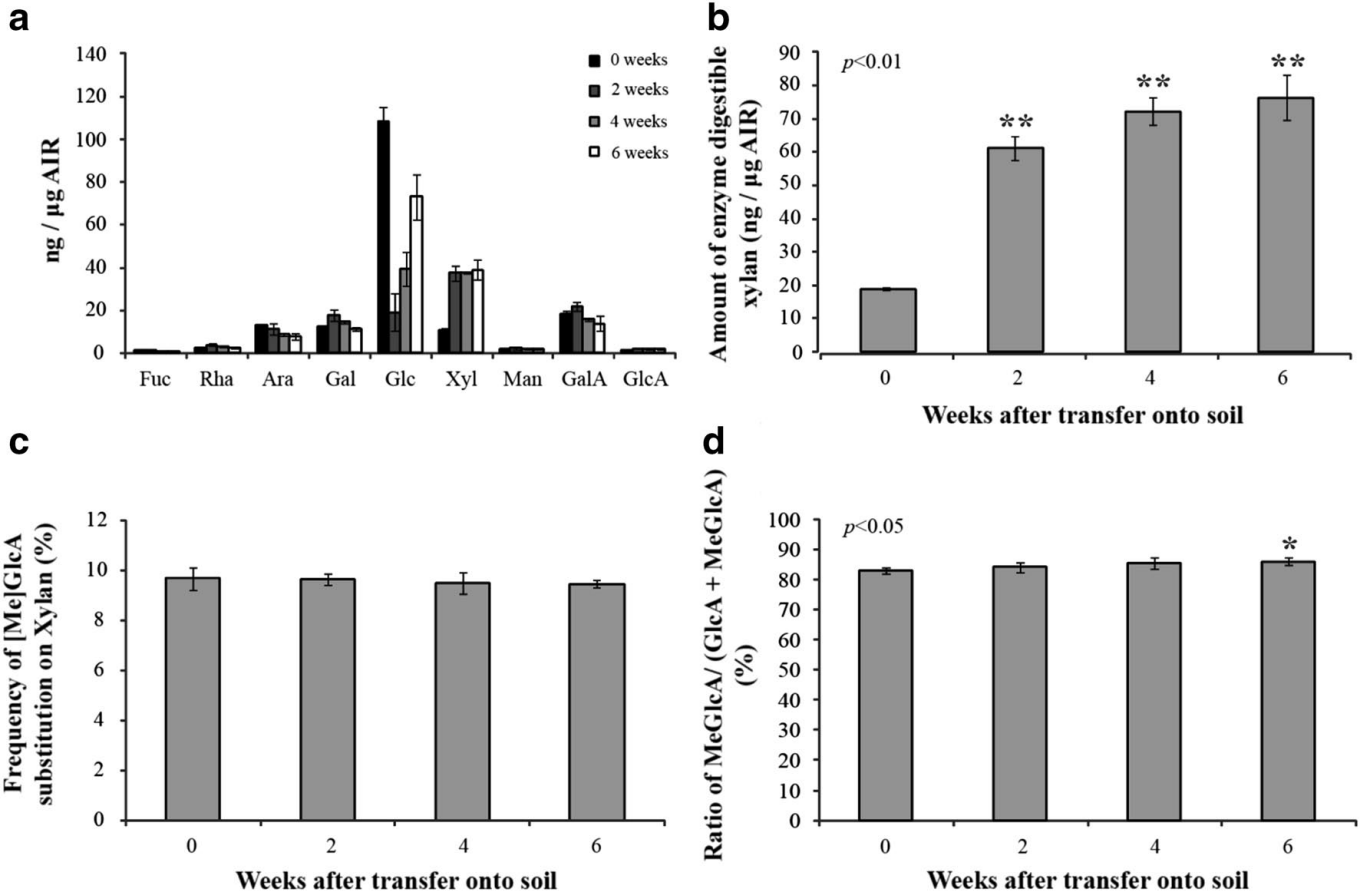
Cell wall composition of internode 3 of developing stems. Averages from three replicate samples of pooled internodes. **a** HPAEC-PAD monosaccharide compositional analysis of willow AIR. **b** Xylan content. **c** Degree of substitution from PACE analysis of willow AIR following endo-xylanase digestion. The oligosaccharide products were quantified using PACE. Values are means of three biological replicates analysed four times ±SD. *Asterisks* indicate significant difference (Student’s *t* test, *P* < 0.01). **d** The amount of methylation of GlcA substitution of xylan was determined by endo-xylanase digestion of willow AIR. The oligosaccharides were quantified using DASH and the frequency of methylation was determined from the quantity of GlcAXyl_4_ relative to the sum of GlcAXyl_4_ and MeGlcAXyl_4_. Values are means of three biological replicates analysed four times ±SD. The *asterisk* indicates significant difference (Student’s *t* test, *P* < 0.05)

The changes in the composition of cell wall polysaccharides in internode 3 were determined by HPAEC-PAD analysis of monosaccharides after acid hydrolysis of AIR (alcohol-insoluble residue) fractions (Fig. 2). Clear changes in molar composition were observed. At zero weeks, the amount of glucose detected by monosaccharide compositional analysis was estimated to be over 100 ng μg^−1^ of AIR which may have been derived mostly from starch since the plants were grown on agar plates supplemented with sucrose. The amount of glucose fell dramatically at 2 weeks, to about 20 ng μg^−1^ of AIR, but then increased again to about 40 ng μg^−1^ of AIR at 4 weeks and 80 ng μg^−1^ of AIR at 6 weeks. The other major change was in xylose, which increased from about 10 ng μg^−1^ of AIR at 0 weeks to 40 ng μg^−1^ of AIR at 2 weeks: this presumably reflects the rapid deposition of xylan in the SCW. Smaller changes were observed in other monosaccharides, including those derived from pectins (rhamnose, fucose, galacturonic acid).

The increase in xylose in Fig. 2a at 2 weeks is attributable to an increase in xylan as shown by quantification of oligosaccharides released by endo-xylanase digestion (Fig. 2b). The total amount of extracted xylan increases from about 20 ng μg^−1^ to over 70 ng μg^−1^ of AIR over the period from 0 to 6 weeks. These analyses also showed that the proportion of xylose (Xyl) residues in the xylan backbone decorated with [Me]GlcA changed little between 0 and 6 weeks, being between 9 and 10 % at all stages. However, the proportion of these GlcA decorations that were methylated increased slightly during development (Fig. 2d); this trend probably continues beyond 6 weeks, since in older willow wood all xylan GlcA is methylated (T.T. and P.D., unpublished).

### Expression of candidate genes for cell wall synthesis

**Fig. 3:**
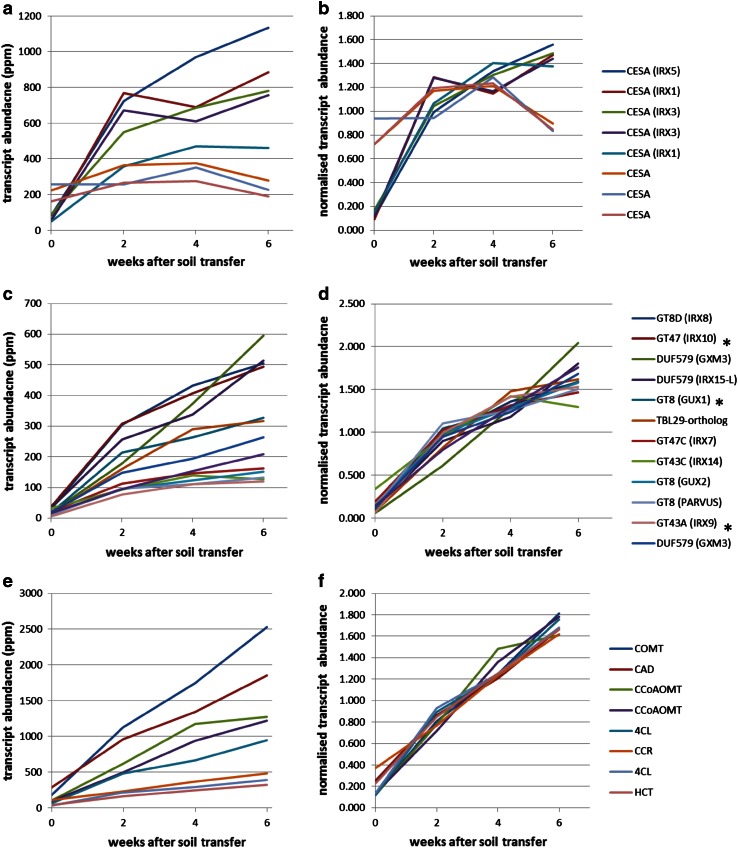
Abundance of transcripts associated with synthesis of cellulose (**a**, **b**), xylan (**c**, **d**) and lignin (**e**, **f**) in internode 3 of developing willow stem. The key shows the gene family with the closest gene homologue in Arabidopsis indicated in *parentheses*; some of these willow transcripts are referred to in the text using “Sp” followed by this Arabidopsis gene name. Some paralogues of transcripts shown in **c** and **d** have been omitted for clarity: a DUF579 IRX15, a GUX1-like and two DUF579 GXM transcripts; these all have profiles more similar in shape to the other xylan transcripts than the GXM transcript shown. A complete list of poplar gene ids to which the willow RNA-Seq reads mapped for the transcripts shown plus lower abundance ones associated with synthesis of cell wall polymers is given in Supplementary Table S1. Three xylan synthesis transcripts selected for in situ analysis are indicated (*asterisk*)

Parallel analyses of the RNA-Seq transcriptome from internode 3 revealed distinctive profiles for cell wall-related transcripts (Fig. 3). Cellulose synthase (CESA) transcripts (Fig. 3a, b) showed the greatest abundance of all the glycosyl transferase (GT) families with homologues of IRX1, IRX3 and IRX5 (SCW-specific CESA genes of Arabidopsis) showing very similar profiles, rising sharply between 0 and 2 weeks and less steeply between 2 and 6 weeks. The other CESA transcripts showed much higher expression at 0 days followed by relatively little change, suggesting that they are involved in the synthesis of cellulose in the primary cell wall, and this is in keeping with the expression patterns of the corresponding Arabidopsis genes. Co-expression with the SCW-specific CESA has been used to identify several GT genes involved in xylan synthesis in Arabidopsis, including IRX7, IRX8, IRX9, IRX10, IRX14 and GUX1, 2 (Brown et al. 2005, 2009; Mortimer et al. 2010) and close homologues of these also showed very similar expression profiles to each other and to the corresponding CESA genes in willow (Fig. 3a–d). In terms of absolute abundance (Fig. 3c), the IRX8 and IRX10 homologues, which are implicated in the synthesis of reducing end oligosaccharide (Zhou et al. 2007) and xylan backbone extension (Brown et al. 2009; Wu et al. 2009), respectively, were the most highly expressed of the xylan-related transcripts. Other cell wall-related genes recently characterised in Arabidopsis are a member of the DUF579 family encoding GlcA methyltransferase (GXM) (Urbanowicz et al. 2012) and a member of the trichome birefringence-like family (TBL29) involved in xylan *O*-acetylation (Xiong et al. 2013). A different clade of DUF579 genes, IRX15 and IRX15-like, are required for normal xylan backbone extension in Arabidopsis (Jensen et al. 2011). All of these genes have close homologues which are abundantly expressed in developing willow internodes; thus candidate transcripts associated with all the main features of SCW xylan structure in dicots are present in willow (summarised in Supplementary Fig. S1). All genes show the same shape of expression profile as the SCW-CESA, with the exception of a GXM transcript [corresponding to poplar gene Potri.004G226800 which is an orthologue of GXM3 (using TAIR nomenclature)]. This GXM3-like transcript is somewhat less abundant early in development and more abundant later compared with the other xylan-related transcripts, the profile being more similar to those of the lignin-related transcripts, which increase approximately linearly with time (Fig. 3e, f).

Some lower abundance transcripts which show similar expression patterns to the xylan-related transcripts in Fig. 3c, d have been omitted for clarity; these are given in Supplementary Table S1, which lists all transcripts similar to genes responsible for synthesis of cellulose, xylan and lignin. This includes transcripts similar to GXM3 and GXM1/2 which have profiles more similar to the other xylan-related transcripts than to the GXM3-like transcript shown in Fig. 3c, d. Functionally redundant paralogues of IRX9, IRX10 and IRX14, called IRX9-L, IRX10-L and IRX14-L are present in Arabidopsis where they are usually expressed at lower levels (Wu et al. 2010). Homologues of all of these minor forms are also present in willow (Supplementary Table S1) and show expression patterns associated with secondary cell walls (defined as co-expression with the IRX3 homologue). It, therefore, appears that both major and minor forms of the SpIRX9, SpIRX10 and SpIRX14 genes are expressed in tissues synthesising xylan for SCW.

A set of 1,445 transcripts from the willow developing stem transcriptome was defined as “SCW-associated” (listed in Supplementary Table S2) by co-expression with the IRX3 orthologue (coefficient of correlation >0.9) [co-expression with IRX3 having been successfully used to identify novel SCW genes in Arabidopsis (Brown et al. 2005)]. A previous transcriptome analysis of secondary growth in poplar (Dharmawardhana et al. 2010) shows considerable overlap with this list and 215 of the poplar orthologues listed in Supplementary Table S2 were also identified as associated with secondary growth. One example is shown in Table 2: a fasciclin-like arabinogalactan protein (FLA). Differential expression of FLA is frequently associated with different types of wood formation. It has recently been shown that an AGP-pectin–xylan proteoglycan exists in Arabidopsis cell walls (Tan et al. 2013). This AGP is not an FLA, but is in the “Classic AGP” family as defined in Showalter et al. (2010) and a transcript in this family is also present in the SCW-associated set (Table 2). Also of interest is the presence of a homologue of a xylosidase from glycosyl hydrolase family 3 (Xiong et al. 2007) suggesting that hydrolysis of xylan is also required as part of SCW deposition. Regulators of SCW are also prevalent in the SCW-associated set; for example, the SND1/NST3 transcription factor has been shown to drive secondary cell wall thickening in Arabidopsis (Mitsuda et al. 2007); KNAT7 appears to be a negative regulator of SCW synthesis in Arabidopsis and poplar but shows co-expression with positive regulators and SCW genes (Li et al. 2012).

**Table 2:** Five willow transcripts discussed in the text selected from set of 1,445 SCW-associated transcripts in Supplementary Table S2

Poplar gene (JGI v3.0)	IRX3 correlation coefficient	Close homologues	Homologue descriptor
Potri.008G012400	1.000	AT5G06390	Fasciclin-like arabinogalactan protein FLA17 (Showalter et al. 2010)
Potri.003G022900	1.000	AT5G64570; A5JTQ3	Bifunctional beta-xylosidase/alpha-l-arabinosidase (Xiong et al. 2007)
Potri.009G092300	0.993	AT2G14890	Classical arabinogalactan protein AGP9C (Showalter et al. 2010)
Potri.011G153300	0.989	AT1G32770	NAC secondary wall thickening promoting factor3/SND1 (Zhong et al. 2006; Mitsuda et al. 2007)
Potri.001G112200; PotriKNAT7	0.945	AT1G62990; AtKNAT7	Class II KNOX negative regulator of SCW synthesis (Li et al. 2012)

The above analysis is all based on the alignment of the willow RNA-seq reads to poplar. We also used de novo assembly to search for willow-specific protein-encoding transcripts (see “Materials and methods”). A total of 403 such contigs (putative transcripts) were found matching with 101 different PFAM domains (Supplementary Table S3); five willow-specific putative glycosyl hydrolases were identified by this method but no glycosyl transferases.

### Cell wall lignification and xylan localisation

**Fig. 4:**
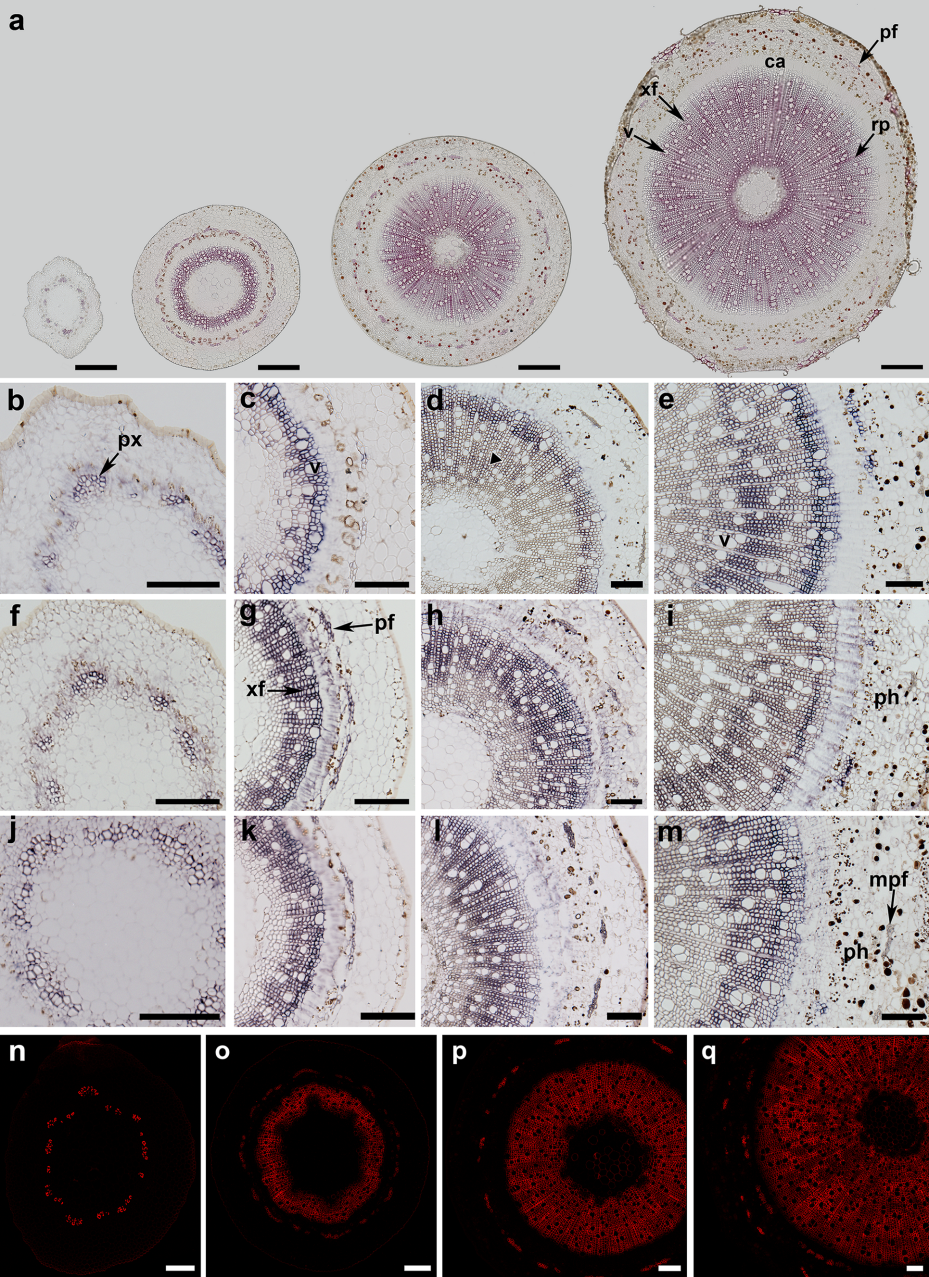
Cell wall development and gene expression in stems at 0, 2, 4 and 6 weeks (from *left* to *right*). **a** Lignification shown by Phloroglucinol–HCl staining (*pink colour*). Staining is localised in the protoxylem at the 0-week stage and maturing secondary xylem and phloem fibres. **b–m** Expression of IRX10 (**b–e**), IRX9 (**f–i**) and GUX1 **(j–m**) transcripts shown by the purple staining occurs mainly in protoxylem (*px*), developing secondary xylem, especially in xylem fibres (*xf*) and in newly formed phloem fibres (*pf*). Expression is generally absent from mature phloem fibres (*mpf*) at the 6-week stage (*arrows*). Also indicated are cambium (*ca*) and ray parenchyma (*rp*). **n–q** Localisation of xylan in cell walls of 0- to 6-week-old stems detected with the LM10 antibody. *Bars* 250 μm (**a**), 100 μm (**b**–**q**)

Phloroglucinol–HCl staining showed that lignification started early in the cell walls of vessels of the primary xylem at week 0. It then proceeded quickly into the secondary xylem, with the exception of the newly formed cell layers adjacent to the cambium in all stages (Fig. 4a). Cell walls of maturing phloem fibre cells also became lignified as early as week 2.

Xylan distribution was determined using the LM10 antibody (Fig. 4n–q), which binds to low- or un-substituted xylan backbone (McCartney et al. 2005). At 0 weeks, strong labelling was present only in the secondary cell walls of the primary xylem (Fig. 4n). From 2 to 6 weeks, the cell walls of all cell types in the secondary xylem exhibited strong fluorescence; however, the signal was weaker in cell walls of newly formed cells close to the cambial zone and no signal was detected in the cell walls of differentiating cells immediately adjacent to the cambium. The signal observed in the cell walls of phloem fibres increased from 2 to 4 weeks (Fig. 4o–p). At 6 weeks (Fig. 4q), mature phloem fibres in the outermost region of the cortex were highly fluorescent, whereas younger newly formed fibres showed weaker signals. In addition, the fluorescence pattern in the secondary xylem was irregular, reflecting the patterns found with in situ hybridization (below). These results are generally consistent with studies of xylan deposition and localisation in 10-year-old poplar (Kim and Daniel 2012) and 3-month-old hybrid aspen (Kim et al. 2012). In the latter study, xylan deposition was found to start earlier and showed more heterogenous labelling of fibres compared with the uniform and strong labelling of secondary vessels and, interestingly, strong labelling of highly substituted xylans was detected during differentiation but then disappeared during pit maturation.

### In situ hybridization of SpIRX10, SpIRX9 and SpGUX1 transcripts

Three RNA probes were designed to determine the expression patterns of homologues of genes related to xylan synthesis. IRX9 and IRX10 are involved in xylan backbone extension in Arabidopsis (Brown et al. 2009; Pena et al. 2007; Wu et al. 2009) and the same role has been demonstrated for PtrIRX9 in poplar (Lee et al. 2011). GUX1 encodes a xylan glucuronosyl transferase in Arabidopsis (Mortimer et al. 2010). The relationship between the willow cDNA sequences cloned to make in situ probes and the Arabidopsis and poplar genes is shown in phylogenetic trees for these clades (Supplementary Fig. S2 and S3). In situ hybridization experiments performed on wax sections revealed similar spatial patterns of expression (shown by the purple staining) for the three genes (Fig. 4b–m). In particular, expression was confined to cells undergoing secondary cell wall deposition. At 0 weeks, signals were detected only in the primary xylem (Fig. 4b, f, j) while at 2 weeks strong expression was observed in several cell layers of the developing secondary xylem next to the cambial zone, but no signal was present in the mature cells close to the pith. Some staining was also observed in phloem fibres (Fig. 4c, f, k). At 4 weeks, expression of transcripts was again mainly confined to the developing secondary xylem (Fig. 4e, l, m) with clear labelling of the ray parenchyma in addition to the xylem fibre cells. At this stage, the pattern of staining was generally more diffuse, extending into the more mature cells in the inner part of the stem (Fig. 4d). Similarly, at 6 weeks the developing secondary xylem exhibited strong signals with all probes (Fig. 4e, i, m). Furthermore, where two layers of phloem fibre bundles were present, expression was observed only in young developing fibres near the cambium and absent from the mature fibres (Fig. 4e, l, m). Most of the sections also exhibited asymmetric staining across the stem with one side showing much weaker signal and/or fewer stained cells. An example is given in Supplementary Fig. S4. Sections treated with control sense probes did not exhibit any staining (Supplementary Fig. S5). The spatial expression patterns of the SpIRX9, SpIRX10 and SpGUX1 were similar for all three transcripts, and similar to those previously reported for the PtrGT43 genes (IRX9 and IRX14 orthologues) in poplar (Lee et al. 2011). Of the three willow transcripts studied by in situ hybridisation, SpIRX10 is the most abundant, and this is the first report of its spatial distribution in trees.

## Discussion

The bud culture system reported here proved to be ideal for the study of early development in willow stems, giving the transition from mostly primary to secondary cell wall synthesis. It provides material which is highly uniform in development, as shown by the small variation, especially in early stages (dry weight, Fig. 1b) and cell wall composition (Fig. 2). The small size lends itself to a high level of replication and shows good reproducibility between experiments. Three integrated levels of study have been carried out on the developing stems: biochemical analyses of cell wall biopolymers, analysis of transcripts by RNA-seq, and visualisation of developmental patterns by light microscopy combined with the use of selective stains and antibodies to reveal polymer distribution and in situ hybridisation to reveal transcript distribution.

As expected, the transition from mostly primary to secondary cell wall synthesis was characterised by a marked increase in xylan of the third internode (Fig. 2b). This increasing trend in xylan accumulation was matched by the rise in abundance of xylan-related transcripts in the same tissue (Fig. 3c, d). Transcripts associated with backbone synthesis (SpIRX9, SpIRX10, SpIRX14) and glucuronyl decoration (SpGUX1,2) were co-regulated and the degree of glucuronyl decoration remained constant through development (Fig. 2c). An interesting trend observed was the small increase in the degree of methylation of glucuronyl decorations (Fig. 2d). This may be attributable to the profile of the most abundant SpGXM3 transcript, which increased relative to other xylan-associated transcripts through development (Fig. 3d) and showed an expression pattern more similar to that of lignin genes (Fig. 3f). To our knowledge, these observations of increasing relative abundance of a GXM transcript and increasing GlcA methylation are new in any tree species. It is not true of Arabidopsis GXM3 which shows close co-expression with xylan transcripts such as IRX10 (as does a second GXM3-like transcript in our willow set; Table S1). The spatial distribution of xylan epitopes (Fig. 4n–q) also matched well to the distribution of the xylan-related SpIRX9, SpIRX10 and SpGUX1 transcripts as revealed by in situ probes (Fig. 4b–m) being confined principally to cells undergoing SCW deposition.

## Conclusion

Regeneration from axillary buds has proved an excellent system to study wood development in willow, providing large numbers of samples with high reproducibility. Using this system, we have been able to produce a RNA-Seq transcriptome resource which has been shown to match well to changes in cell wall composition and demonstrated its utility in examining the spatial distribution of composition and gene expression. This RNA-Seq resource (deposited in the ArrayExpress database, Accession number E-MTAB-2166) can be mined to determine the abundances and expression profiles of transcripts from candidate genes for compositional traits as these emerge, e.g. from genetic studies on willow.

## Electronic supplementary material

Below is the link to the electronic supplementary material.

Supplementary material 1 (XLSX 28 kb)

Supplementary material 2 (XLSX 407 kb)

Supplementary material 3 (XLSX 15 kb)

Supplementary material 4 (PPT 6184 kb)

## Abbreviations

AIR: Alcohol insoluble residue
APTS: 8-Aminopyrene-1,3,6-trisulfonic acid
CTAB: Hexadecyltrimethylammonium bromide
DIG: Digoxigen
EDTA: Ethylene diaminetetra-acetic acid
DEPC: Diethylpyrocarbonate
GT: Glycosyl transferase
IBA: Indole-3-butyric acid
IRX: Irregular xylem
NBT/BCIP: Nitro blue tetrazolium chloride/5-bromo-4-chloro-3-indolyl phosphate toluidine salt
SCW: Secondary cell walls
