# Supplementary material for: Secondary cell wall composition and candidate gene expression in developing willow (Salix purpurea) stems
Source: Planta. 2014 Feb 7;239(5):1041–53. doi: 10.1007/s00425-014-2034-1 (PMC3997797; doi:10.1007/s00425-014-2034-1)
Supplement: Supplementary file 4 — Supplementary material 4 (PPT 6184 kb) [file 425_2014_2034_MOESM4_ESM.ppt]

## Slide 1
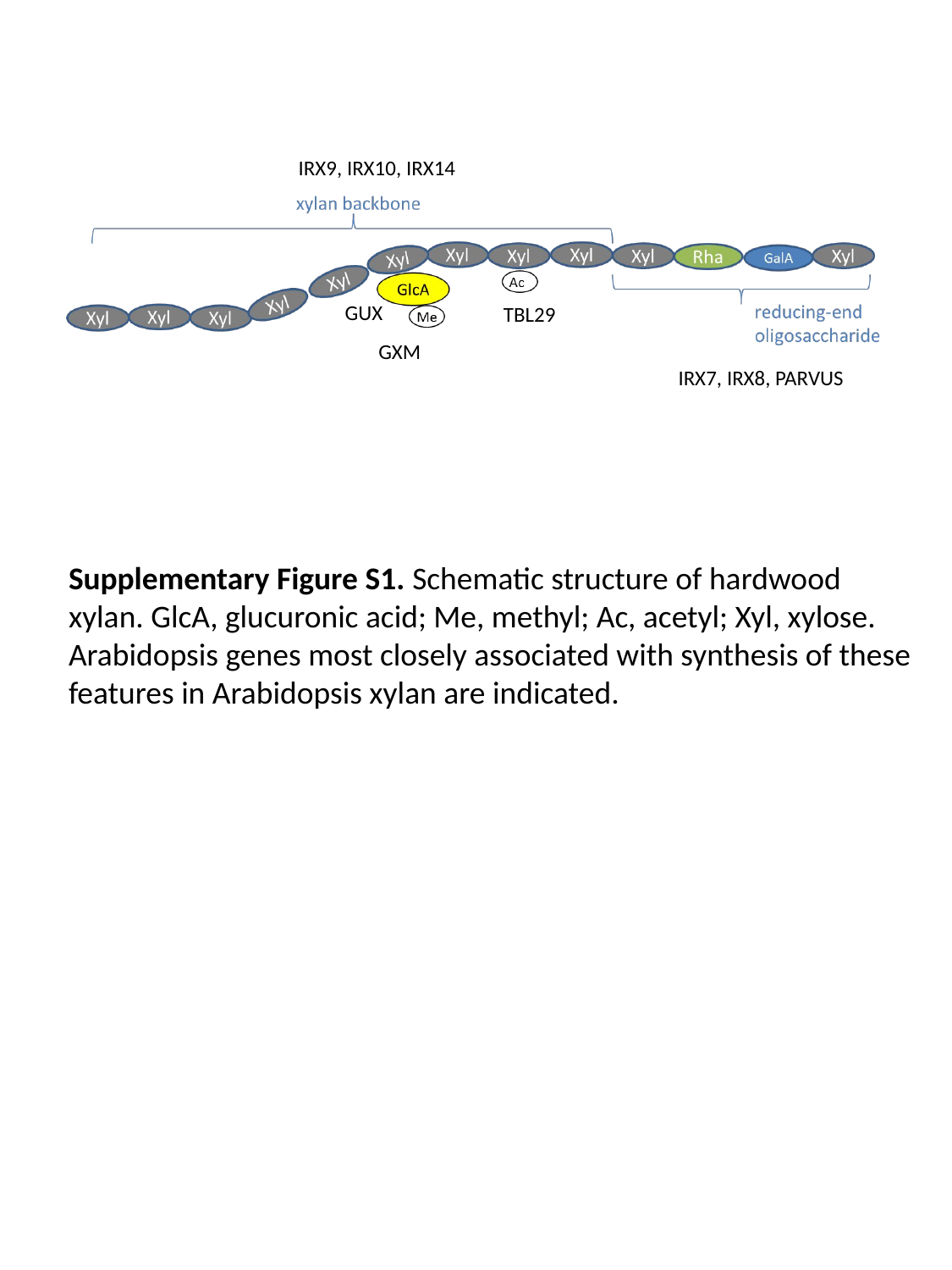

IRX9, IRX10, IRX14
GUX
TBL29
GXM
IRX7, IRX8, PARVUS
Supplementary Figure S1. Schematic structure of hardwood xylan. GlcA, glucuronic acid; Me, methyl; Ac, acetyl; Xyl, xylose. Arabidopsis genes most closely associated with synthesis of these features in Arabidopsis xylan are indicated.

## Slide 2
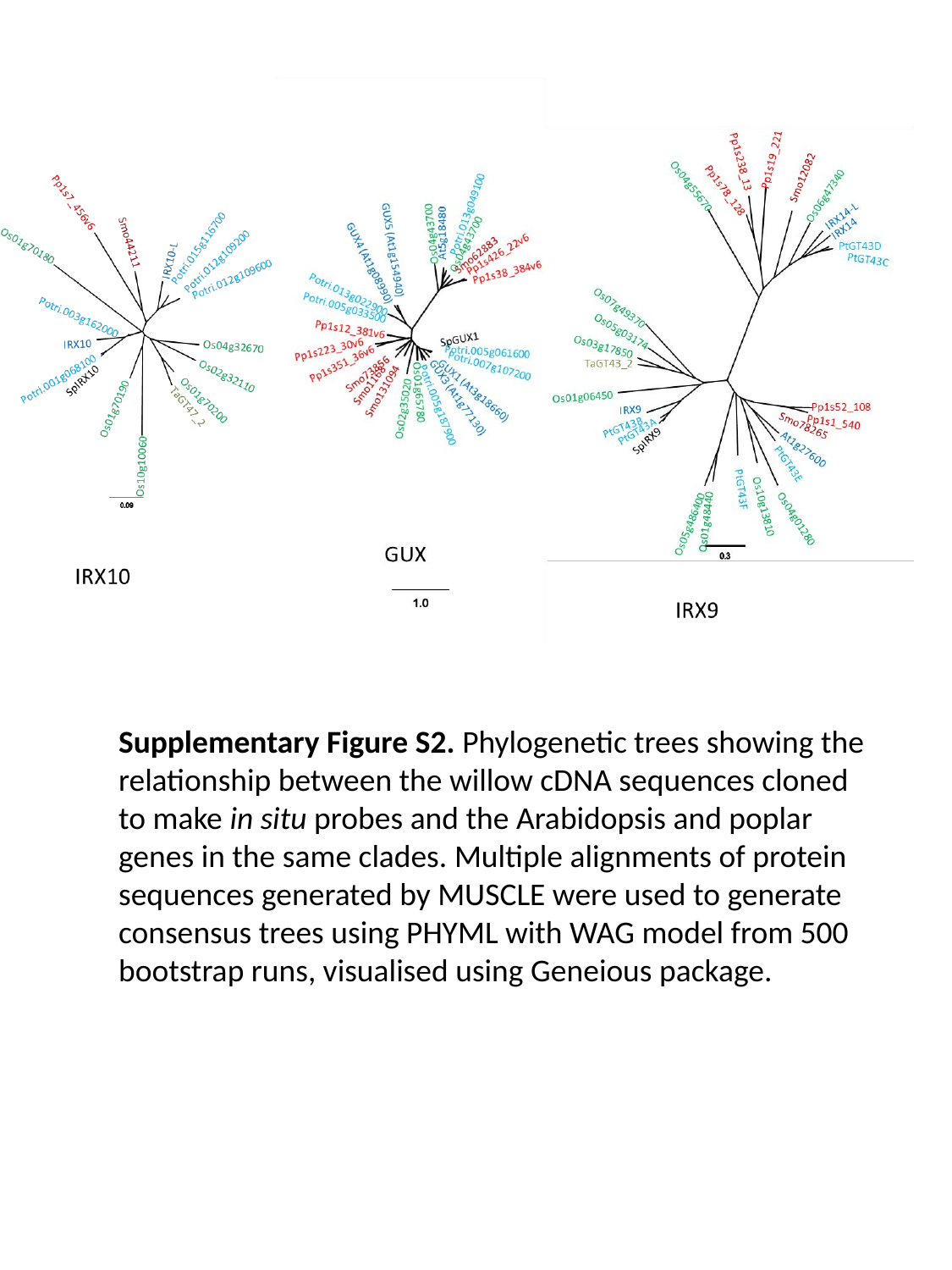

Supplementary Figure S2. Phylogenetic trees showing the relationship between the willow cDNA sequences cloned to make in situ probes and the Arabidopsis and poplar genes in the same clades. Multiple alignments of protein sequences generated by MUSCLE were used to generate consensus trees using PHYML with WAG model from 500 bootstrap runs, visualised using Geneious package.

## Slide 3
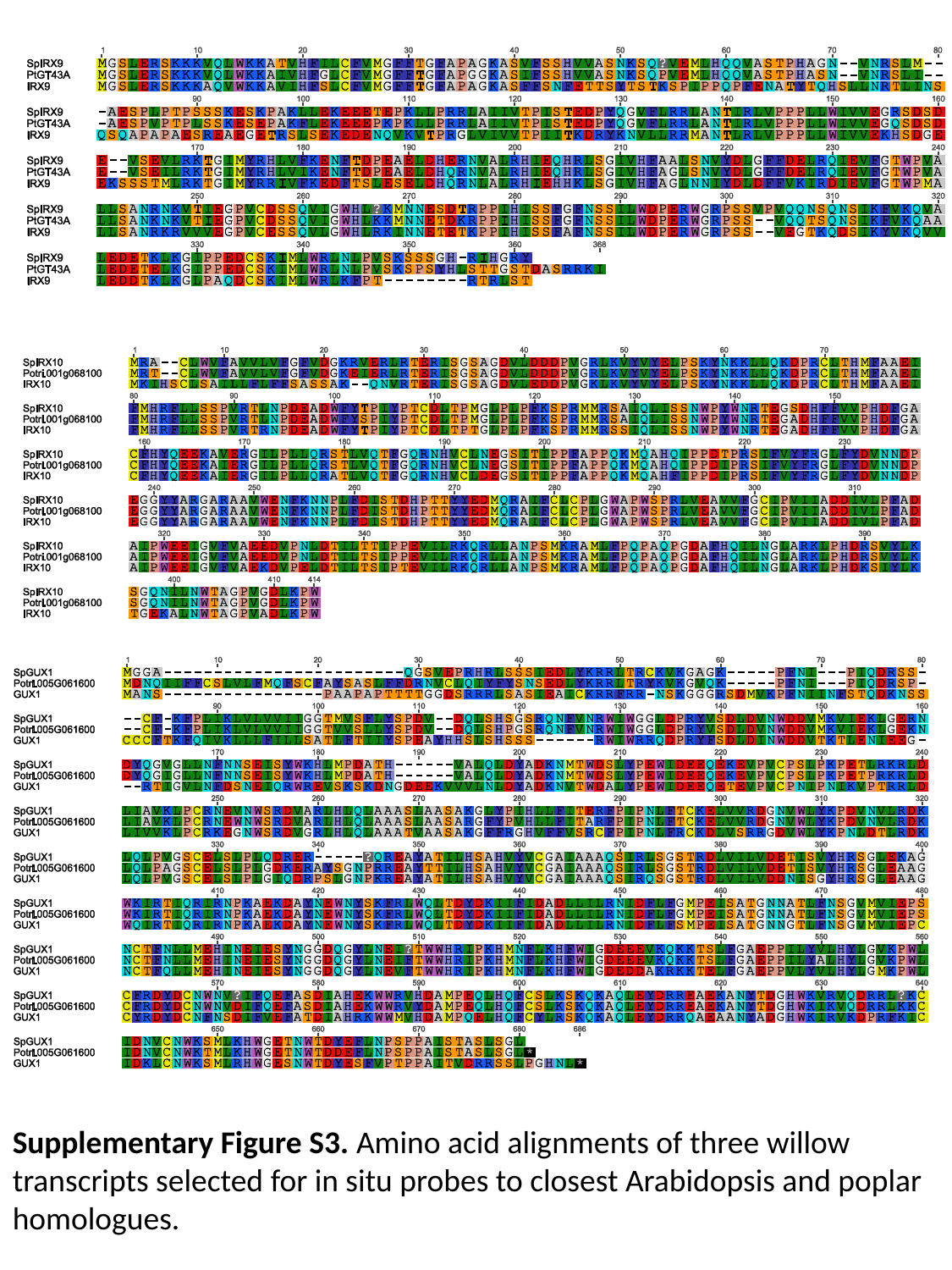

Supplementary Figure S3. Amino acid alignments of three willow transcripts selected for in situ probes to closest Arabidopsis and poplar homologues.

## Slide 4
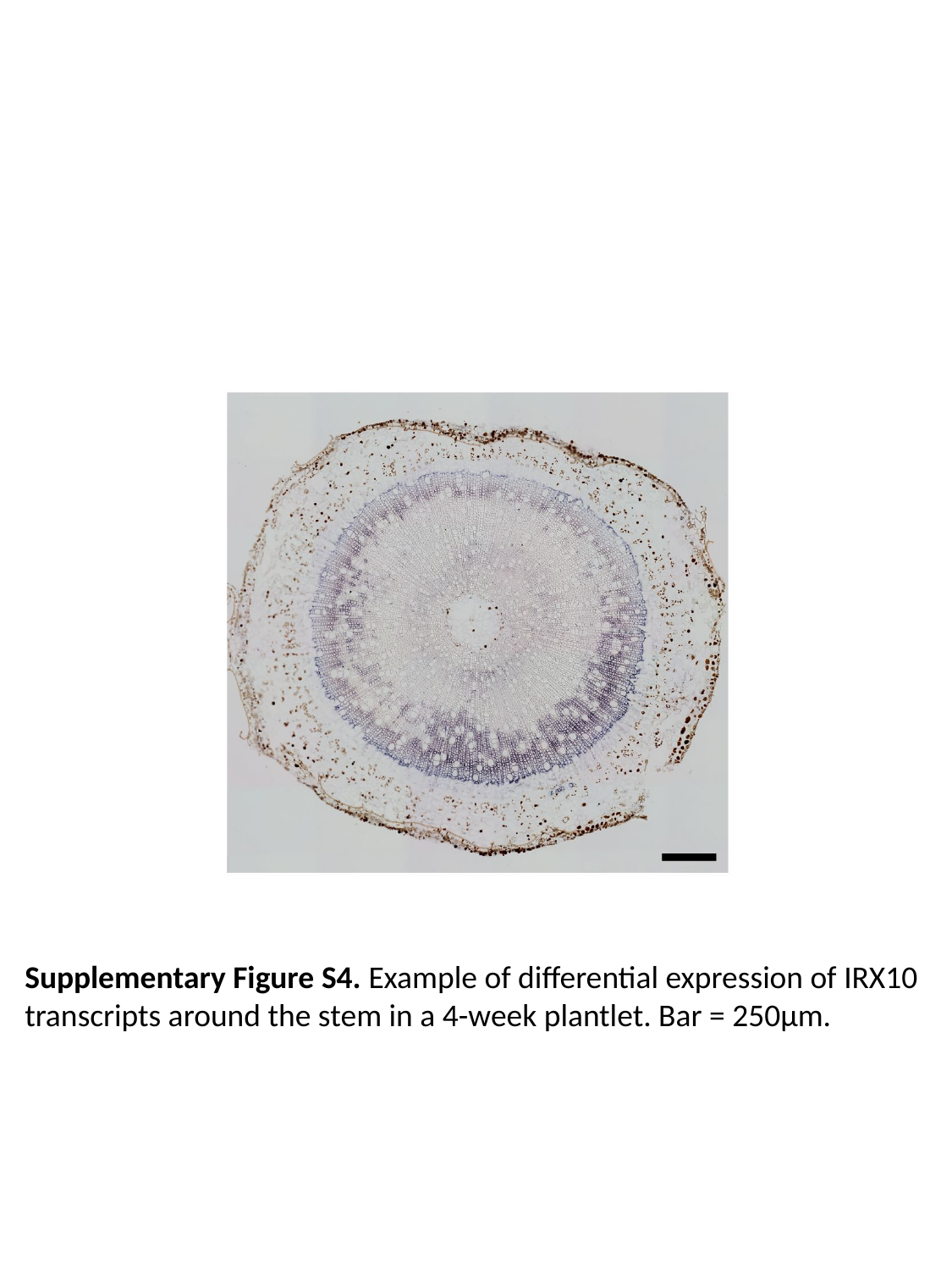

Supplementary Figure S4. Example of differential expression of IRX10 transcripts around the stem in a 4-week plantlet. Bar = 250µm.

## Slide 5
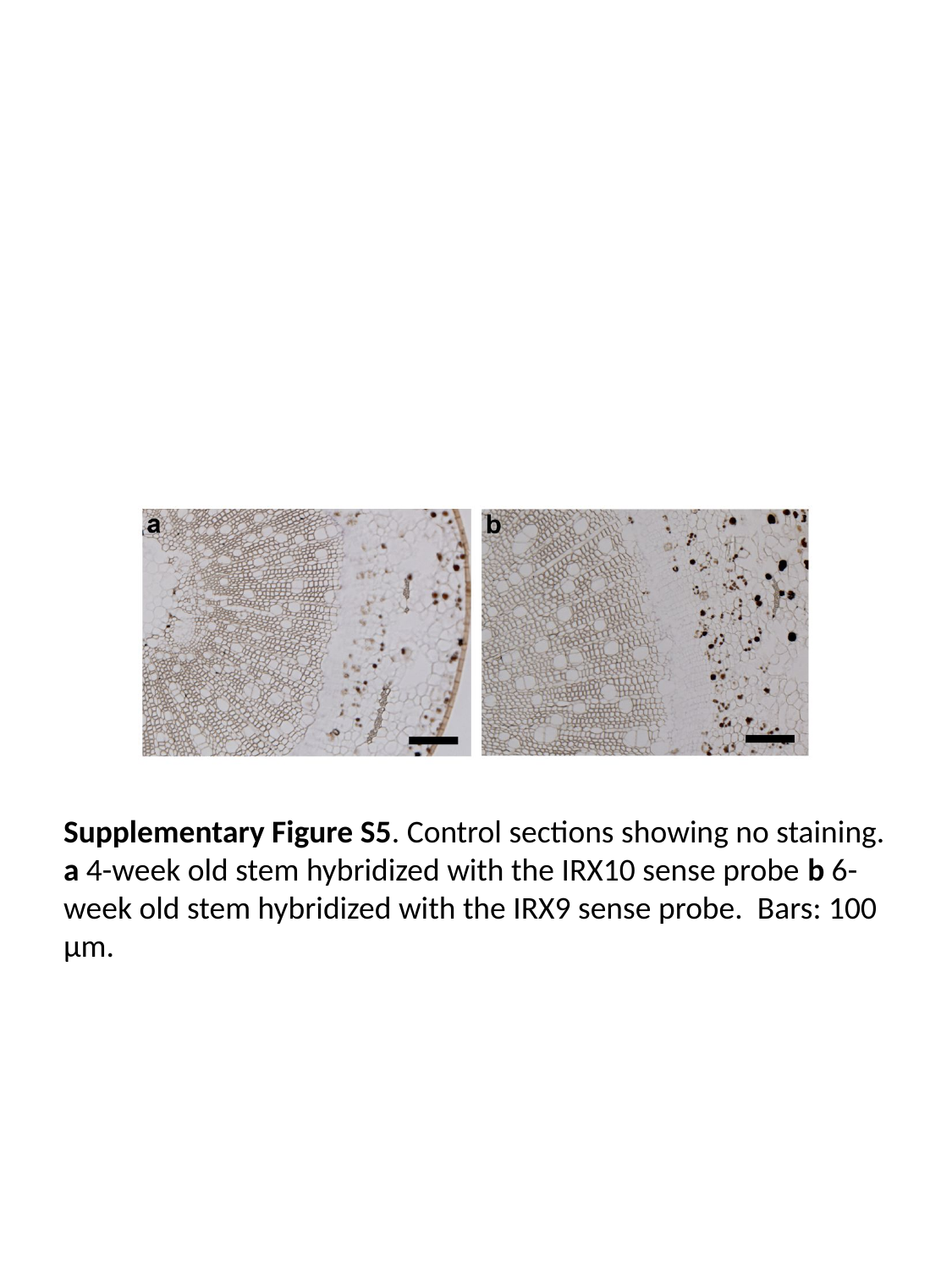

Supplementary Figure S5. Control sections showing no staining. a 4-week old stem hybridized with the IRX10 sense probe b 6-week old stem hybridized with the IRX9 sense probe. Bars: 100 µm.
